# A Treatise on Pyrosis Idiopathica, or Water-Brash, as Contrasted with Certain Forms of Indigestion and Organic Lesions of the Abdominal Organs; Together with the Remedies, Dietetic and Medicinal

**Published:** 1841-10

**Authors:** 


### ART. XVI.

*A Treatise on Pyrosis Idiopathica, or Water-brash, as contrasted with certain forms of Indigestion and Organic Lesions of the Abdominal Organs; together with the Remedies, Dietetic and Medicinal.* By THOMAS WEST, M.D.—*London,* 1841. 8vo, pp. 108.

THE theory of Pyrosis, advanced in the present volume, appears (p. 24) to have been suggested by Andral. According to Dr. West, pyrosis is a disease of debility of the nervous system generally, but more particularly of the nervous apparatus of the stomach and first passages; which debility, partial or general, may, as we shall by and bye see, be produced by a variety of causes. The pyrotic discharge he regards as a sort of passive serous "percolation" from "the terminal exhalants of the œsophageal, stomachic, and intestinal mucous membrane," very analogous to the colliquative sweats that occur in states of great exhaustion; or to the internal dropsies, anasarcous or serous (i.e. in serous cavities), resulting from the same causes. Indeed, the author proposes to designate pyrosis, "a dropsy of the stomach:" and quotes Andral to show that that eminent pathologist had already pointed out certain analogies between the two diseases so named.

"It may be objected that it is a solecism to name any collection of fluid a dropsy, if not occurring in a serous or close cavity. The objection is plausible only at first sight: it will not bear reflection. Make your close cavity an open one by paracentesis, and does your dropsy cease? Have you not dropsy of the womb recognized by the older nosologists? Open a hydrocele, and is it not yet a dropsy, until you have altered the action on the surface of the tunic so as to obviate further effusion? But it may be said that the effused fluid in a pyrosis is sure to be discharged by vomiting or rumination. This also is doubtful. In some cases the effusion collects until it has produced a sudden and visible distension of the stomach itself, and gradually passes off (as may be inferred) through the pylorus into the intestines," &c. (p. 71.)

He elsewhere remarks (p. 9,) . . . "where the weakness is limited to the serous structure of the stomach itself, we have pyrosis only; but in protracted and inveterate cases, as the debility extends to the rest of the serous system, we have dropsy of cavities, or general dropsy."

These views, if correct, would give to water-brash a degree of importance which it has not been customary to assign to it. We must, however, remark that dropsy, as a sequel of simple pyrosis, and as connected with it, is exceedingly rare; although, as ensuing on chronic derangement of the chylo-poietic viscera, of which pyrosis may have been a prominent *symptom*, it is not uncommon. Now, it would have been satisfactory to have found Dr. West expressing himself clearly and consistently on this subject; but we are at some loss to reconcile the following statements: "Pyrosis then, in our view, is only an *aggravation of one of the common symptoms* of derangement of the stomach." In like manner, at page 33, he heads a paragraph thus: "Pyrosis may be merely *symptomatic* of pregnancy." While at page 73, he commences the chapter on treatment by saying: "Is pyrosis idiopathic or symptomatic? I answer confidently, the disease of which I am treating is decidedly idiopathic . . . If this be not an idiopathic malady, neither is cholera, nor dropsy, nor scurvy." These statements appear to us to be discrepant. In our opinion pyrosis has no more direct connexion with dropsy than any other affection by which general debility is indicated. Dr. West's meaning in the above passages probably is, that while pyrosis is occasionally symptomatic, it *may* be, in other cases, idiopathic.

In one way, indeed, pyrosis may have a somewhat directer relation with the other more formidable disease. We have seen cases in which the daily discharge of pyrotic fluid was by no means inconsiderable; and Andral mentions a case in which it amounted to four pints. In such cases, should the discharge by any cause be interrupted, it might, by being thrown on some other surface or channel,—by "suddenly changing its character for deposition in close cavities," to use Andral's words as quoted by Dr. West, give rise to forms of dropsy.

Dr. West's account of the source of the pyrotic fluid is at least questionable. At pp. 6, 32, and elsewhere, he invariably assumes that it undoubtedly issues from what he calls the terminal serous vessels. We think it accords more with analogy to suppose that a secretion, possessing such physical characters (acridity, acerbity, &c.,) as that of water-brash often does, is more likely to be a glandular secretion, than one from terminal exhalants. It is very probable that the fluid given out in the disease designated by Andral, "Follicular Gastric Dyspepsia," has the origin which he conjectures, and which is implied by its name. We are disposed, on the other hand, to think it probable that the pyrotic fluid comes from those glands described by Dr. Boyd, in his Inaugural Essay on the Structure of the Mucous Membrane of the Stomach, of which a good engraving is given in Müller's Physiology, as translated by Baly. We have no reason to suppose that serous vessels have any power of modifying the fluid which passes from them. And as it is impossible to believe that a fluid so acrid, acid, &c. as the pyrotic occasionally is, exists free in the blood, this makes it more probable that it is *elaborated* from the latter by some glandular apparatus, than that it merely "percolates" from terminal exhalant vessels. We are rather confirmed than shaken in this view, by Dr. West's reasonings, at pp. 24-5, where he remarks that this dropsy of the stomach may "suddenly exchange its character for depositing in close cavities, that the reverse may happen; that hydrothorax may terminate by a sudden serous discharge from the air-passages; hydrocephalus by inordinate discharge from the skin, kidney, or bowels. .... Who doubts," he continues, "that in all these cases, the discharge is the same liquid which would have gone off by sweats, or which would otherwise have remained shut up in the close cavities?" Granted: but the serous discharges referred to are wholly unaccompanied by anything analogous to the acute cardialgia which generally characterizes pyrosis, with an acrid discharge: and which seems to prove that the nature of the cases is distinct.

To pass now to practical matters, Dr. West's account of the symptoms of pyrosis is very accurate:

"The rumination of the liquor is preceded by anxiety, a sense of uneasiness, cardialgia, pain or burning heat in the epigastrium, which mark the period of secretion; and the ejection of the fluid is always followed by temporary relief. The fluid ejected is usually copious, transparent, hot. Sometimes it is acrid and corrosive, leaving the tongue rough, as if an acid had passed over it: at other times it is insipid or slightly brackish; in rarer instances, of various shades of colour. In inveterate cases, the rumination runs into vomiting, and sometimes into true erythematic gastritis. In these cases, after the inflammation of the stomach has been subdued, the pyrosis still remains to be dealt with, and is, for the most part, more unmanageable." (p. 2.)

His account of the usual causes is also very correct:

"Among the most frequent causes are, deficiency of clothing in a damp and cold atmosphere; error in diet; coarse barley-bread; salt fat pork and bacon; with an undue admixture of coarse vegetable matter, such as greens and potatoes, &c.; the habitual ingestion of ardent spirits of bad quality, or in undue quantity; smoking, snuffing, habitual depraved positions of the body, as in cases of shoemakers, tailors, ribbon-weavers, &c., a poor and scanty diet, with irregularity of meals; hot tea drinking in excess. In other cases, I find none of these or such like causes, but rather that the malady may be referred to the *vires vitæ* having been shattered by long and incessant labour in approaching old age; or in women by rapid and frequent breeding; and in some I have been disposed to refer it to excessive dosing with empirical remedies, to frequent mercurialism, to the abuse of purgatives, to a depraved habit of voraciously eating or rather bolting of food. In other cases, I have satisfactorily referred the mischief to excessive constipation of the lower bowels." &c. (p. 10.)

In pointing out the diagnostic distinctions between follicular gastrorrhea or stomach-gleet and pyrosis, Dr. West thus sums up (p. 32): "I would infer, therefore, that pyrosis may be readily distinguished from stomach-gleet, by the burning pain, by the hot serous secretion, and by its not being complicated with catarrh, nor with humoral asthma, nor with rheumatism." With regard to the alleged absence of burning pain, in stomach-gleet, it does not correspond with our experience; it is not invariably absent. This particular in the diagnosis is therefore fallacious. We have remarked pain or gnawing, and that of a very acute character, in cases of pure follicular gastrorrhea; nor can we theoretically understand why this may not occasionally be; seeing that stomach-gleet is sometimes owing to the same causes, local irritation, or disturbed innervation, as pyrosis itself is.

The pathological post-mortem appearances are not decisive of the origin and nature of pyrosis.

Passing over the remarks at pp. 72, et seq., the object of which is to establish what we are constrained to regard as an entirely fanciful analogy between Asiatic cholera and pyrosis—the same effects being supposed by the author to be suddenly produced and in an extreme degree in the former disease as are induced slowly in the latter,—we come to the treatment of the malady, which, as stated by Dr. West, seems judicious and successful.

The general indications (p. 104) are thus given : "1st, To provide azotic food in order that the blood of the patient may be more highly animalized ; 2d, to increase steadily the vigour and heat of the circulating system, especially in the capillaries of the surface ; 3dly, to give new tone and fresh impulse to the absorbent system." In preceding parts of his treatise, Dr. West points out the necessity of these means. We willingly quote the following observations, bearing, as they do, on a too common error of the public, and sometimes even of practitioners ; namely, that of expecting from mere medicinal means that relief which can only be obtained from proper diet or regimen.

"We may try to rectify secretions, to brace up and fortify the stomach ; but unless we can supply good wholesome food, animal and vegetable, together with warm clothing, a dry habitation, and the peace of mind which is the attendant upon a cheerful countenance, or at least a good hope of a continuance of a comfortable 'daily bread,' all our measures will fall short of relief, and our pills and potions, our chemicals and our galenicals, will be a miserable mockery, a wretched satire upon that half science and half art, which Cicero denominated 'God's second cause of health.' Now, as a deficiency of wholesome food, of fuel, and of good clothing, is a common cause of pyrosis, so it is also, of anasarca. To what and whither shall we retreat for these necessaries of life, in a variable and fickle climate? Do our patients find these comforts in our provincial dispensaries? We bind their stomachs with bismuth or with bark, and we send them back to beggary." (p. 19.)

Besides its merely superior nutritive qualities, Dr. West assigns another reason for the employment of azotic food (p. 57): "That non-azotic food will not eliminate the same quantity of heat in the human body as aliment of a more generous quality is a fact admitted and implied in establishing our indications for the treatment of disease. We avoid strongly azotized materials in fevers, &c. . . . When I see the powers of the body, the pneumo-gastric energies especially, giving way as in pyrotic patients, under the use of an aliment seriously deficient in azote, and when I see such patients gradually becoming lower in their temperature," &c. Dr. West notices that the frequency of the cases in country districts augments in winter and diminishes in summer, which he attributes to the more plentiful supply of milk in the latter season. A milk and bread diet and shelter often suffice for cure with patients accustomed to a poor and scanty mode of living, and suffering from exposure. The bowels should be regulated by tonic aperients. Dr. West appends some useful prescriptions in the various forms, liquid, pilular, and pulverulent.

This monograph, though containing a good deal of extraneous matter and untenable theory, will probably be useful, not only from its own intrinsic merits (our opinion of which may be gathered from the tenor of the preceding remarks), but also from its drawing attention to a somewhat obscure and neglected subject.

